# Comparing the use of the Optos Silverstone widefield imaging system with ultrasound B-scanning for the assessment of choroidal naevi

**DOI:** 10.1038/s41433-026-04437-w

**Published:** 2026-03-27

**Authors:** Laura N. Cushley, Ryan Murdock, Tunde Peto, Anjanette McCaw, Richard Best, Giuliana Silvestri

**Affiliations:** 1 Centre for Public Health, Queen’s University Belfast, Belfast, UK; 2 Department of Ophthalmology, Belfast Health and Social Care Trust, Belfast, UK

**Keywords:** Physical examination, Epidemiology

Choroidal naevi are present in 5–7% of the United Kingdom population, with a small number of around 6 per million population per year developing a cancerous ocular melanoma [1]. Historically, enlargement of the lesion was considered one of the most reliable indicators of malignancy [2, 3] alongside other risk factors, thickness, presence of orange pigment, absence of drusen, subretinal fluid, diameter and melanoma hollowness [4]. The current gold standard of care is slit lamp assessment and B-scan ultrasound however recent studies show multimodal imaging is able to adequately detect risk factors associated with choroidal naevi.

New patients referred to the choroidal naevi clinic were invited to attend an imaging clinic for assessment. All patients were imaged using Optos Silverstone following a protocol which included:

- Colour widefield imaging,
- Fundus autofluorescence widefield image
- A macula raster scan
- A raster scan, volume scan, HD volume scan, extended line scan or line scan of the naevus
- A B-scan was completed by a doctor on two thirds of patients.

All images were graded and measured by an experienced retinal grader and consultant ophthalmologist. Lesion width and height were measured on colour imaging and area was calculated as shown in Figure 1. Thickness of the lesion was measured on OCT from the top of the lesion to Bruch’s membrane. Naevi were assigned a MOLEs score [5] and data were analysed using SPSS (Version 29) for frequency analysis.

**Fig. 1:**
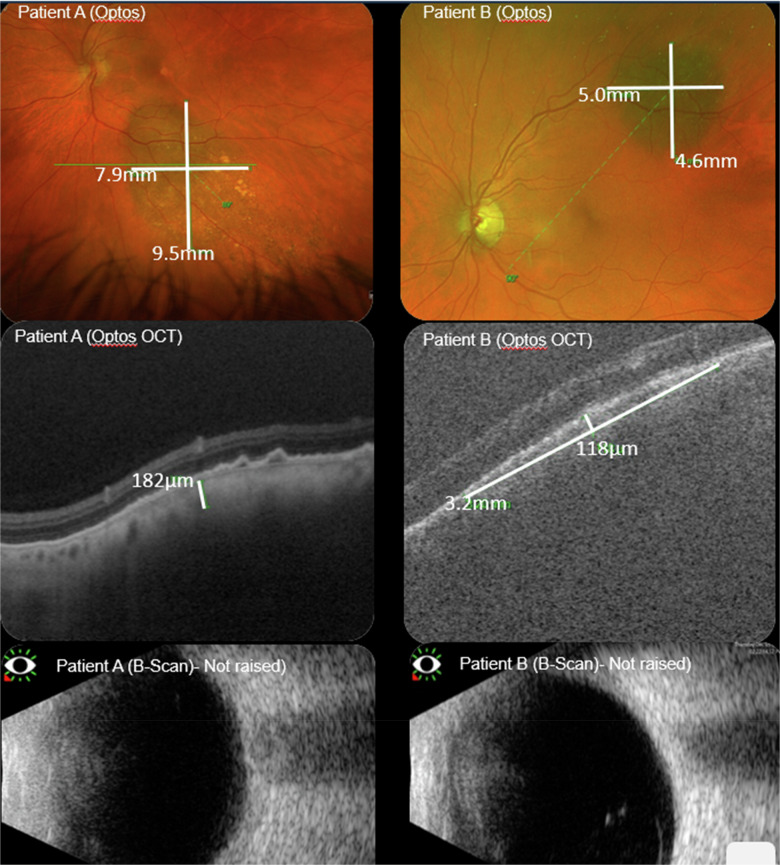
Examples of patient images and scans. These images and scans include Colour, OCT and B-Scans showing lesions raised on OCT but not on B-Scan.

A total of 61 new patients were invited and 3 did not attend their appointment. There were 23 males (39.7%) and 35 females (60.3%), and the mean age was 56.4 years (range 20–84 years). Five patients had a naevus in both eyes equalling a total of 63 eyes.

Of the 63 eyes, 38 had a naevus present (60.3%). Of the 38, (5.2%) 2 naevi were unable to be properly assessed using imaging alone due to being in the very far periphery and 1 (2.6%) due to the presence of a cataract, leaving a total of 35 for assessment Information on naevus location in the retina can be found in Table 1.

**Table 1:** Naevus retinal location.

Area of Retina	Number (%)
Macula	1 (2.8%)
Arcades	16 (45.7%)
Optic Disc	2 (5.7%)
Near Optic Disc	2 (5.7%)
Periphery	12 (34.3%)
Far Periphery	2 (5.7%)

A majority had a MOLES score of 0 (74.3%), with a few [5] having a MOLES score of 1 14.3%) and 2 (8.6%), only 1 (2.9%) had a MOLES score of 4. The mean area was 11 mm^2^, median 4.8 mm^2^ (range 1.17–81.9 mm^2^).

Twenty-five naevi were also assessed using B-Scan ultrasound (71.4%), and 11 (44%) were identified as being raised on OCT with only 1 identified as raised on B-Scan ultrasound (4%). This showed that there was a 100% agreement in terms of OCT vs B-Scan ultrasound. The 10 other naevi detected as raised by OCT and not B-scan ultrasound were all below the threshold of 1 mm, the sensitivity of the average B-Scan ultrasound.

Only one patient was referred to the national oncology centre, and the lesion was determined to be non-cancerous, all others will continue to be monitored.

This study shows that OCT can be used to effectively assess choroidal naevi and refine who has high risk features warranting a full clinical examination and can be used to effectively monitor naevus progression.

## Data Availability

Due to the sensitive nature of the data and the presence of clinical information, the datasets cannot be shared publicly.
